# Lung Ultrasound Score as a Predictor of Mortality in Patients With COVID-19

**DOI:** 10.3389/fcvm.2021.633539

**Published:** 2021-05-25

**Authors:** Zhenxing Sun, Ziming Zhang, Jie Liu, Yue Song, Shi Qiao, Yilian Duan, Haiyan Cao, Yuji Xie, Rui Wang, Wen Zhang, Manjie You, Cheng Yu, Li Ji, Chunyan Cao, Jing Wang, Yali Yang, Qing Lv, Hongbo Wang, Haotian Gu, Mingxing Xie

**Affiliations:** ^1^Department of Ultrasound Medicine, Union Hospital, Tongji Medical College, Huazhong University of Science and Technology, Wuhan, China; ^2^Hubei Province Key Laboratory of Molecular Imaging, Wuhan, China; ^3^Department of Gynecology and Obstetrics, Union Hospital, Tongji Medical College, Huazhong University of Science and Technology, Wuhan, China; ^4^British Heart Foundation Centre of Research Excellence, King's College London, London, United Kingdom

**Keywords:** COVID-19, SARS-CoV-2, lung ultrasound score, mortality, prognosis

## Abstract

**Background:** Lung injury is a common condition among hospitalized patients with coronavirus disease 2019 (COVID-19). However, whether lung ultrasound (LUS) score predicts all-cause mortality in patients with COVID-19 is unknown. The aim of the present study was to explore the predictive value of lung ultrasound score for mortality in patients with COVID-19.

**Methods:** Patients with COVID-19 who underwent lung ultrasound were prospectively enrolled from three hospitals in Wuhan, China between February 2020 and March 2020. Demographic, clinical, and laboratory data were collected from digital patient records. Lung ultrasound scores were analyzed offline by two observers. Primary outcome was in-hospital mortality.

**Results:** Of the 402 patients, 318 (79.1%) had abnormal lung ultrasound. Compared with survivors (*n* = 360), non-survivors (*n* = 42) presented with more B2 lines, pleural line abnormalities, pulmonary consolidation, and pleural effusion (all *p* < 0.05). Moreover, non-survivors had higher global and anterolateral lung ultrasound score than survivors. In the receiver operating characteristic analysis, areas under the curve were 0.936 and 0.913 for global and anterolateral lung ultrasound score, respectively. A cutoff value of 15 for global lung ultrasound score had a sensitivity of 92.9% and specificity of 85.3%, and 9 for anterolateral score had a sensitivity of 88.1% and specificity of 83.3% for prediction of death. Kaplan–Meier analysis showed that both global and anterolateral scores were strong predictors of death (both *p* < 0.001). Multivariate Cox regression analysis showed that global lung ultrasound score was an independent predictor (hazard ratio, 1.08; 95% confidence interval, 1.01–1.16; *p* = 0.03) of death together with age, male sex, C-reactive protein, and creatine kinase-myocardial band.

**Conclusion:** Lung ultrasound score as a semiquantitative tool can be easily measured by bedside lung ultrasound. It is a powerful predictor of in-hospital mortality and may play a crucial role in risk stratification of patients with COVID-19.

## Background

The coronavirus disease 2019 (COVID-19) is a newly recognized infectious disease caused by the severe acute respiratory syndrome coronavirus 2 (SARS-CoV-2). Although chest computed tomography (CT) has been regarded as an important diagnostic tool for COVID-19 diagnosis (1), it is limited by high cost, radiation exposure, infection control challenges, and lack of continuous monitoring, particularly for critically ill patients (2). Lung ultrasound (LUS), with the advantage of being non-invasive, low cost, and radiation free, has been increasingly used as a bed-side tool for evaluation and monitoring of lung diseases, particularly in the intensive care unit (ICU) (2, 3). It was found to have high accuracy in diagnosing viral community-acquired pneumonia with 94% sensitivity and 89% specificity for the detection of viral pneumonia in symptomatic patients (4). Global LUS score, a semiquantitative numerical score of lung aeration across 12 lung regions, has been shown as a useful tool to diagnose acute respiratory distress syndrome (ARDS) (5).

We therefore hypothesized that LUS score may play an important role in detecting lung lesions and optimizing risk stratification in patients with COVID-19. To test this hypothesis, LUS images in patients prospectively recruited from three hospitals in Wuhan, China were analyzed to evaluate the prognostic value of LUS score for in-hospital mortality in patients with COVID-19.

## Materials and Methods

### Patient Population

Patients with confirmed COVID-19 who underwent lung ultrasound were consecutively recruited from the West Branch of Wuhan Union Hospital, Cancer Centre of Union Hospital, and Jianghan Mobile Cabin Hospital Wuhan, China between February 6, 2020 and March 15, 2020. The study was approved by the ethics committee, Union Hospital, Tongji Medical College, Huazhong University of Science and Technology (No. 20200021). Written informed consent was waived because of the unprecedented nature of COVID-19 pandemic.

Inclusion criteria were age ≥18 years and confirmed COVID-19. Exclusion criteria were incomplete image acquisition, missing clinical data, and cardiac failure causing cardiogenic pulmonary oedema.

Demographic, clinical history, comorbidities, laboratory data, and outcomes of all patients were obtained from electronic medical records (Dthealth Medical Systems CO, Tianjin, China). Primary outcome was all-cause mortality. All patients were followed up until April 7, 2020 when the last patient in the study was discharged.

### Lung Ultrasound

LUS examinations were performed by nine qualified ultrasound doctors using Mindray M9 potable ultrasound machines (Mindray Bio-medical electronics Co, Shenzhen, China) with 1- to 5- MHz convex probes. LUS consisted of 12 different regions (two anterior, two lateral, and two posterior thoracic regions) (Supplementary Figure 1) as previously described (6). All video files were recorded in a hospital local archive and were interpreted and scored offline by two experienced observers within 24 h of LUS examinations who were blinded to the clinical data and outcomes. In case of disagreement between observers, the two observers agreed by consensus on the LUS score.

**Figure 1:**
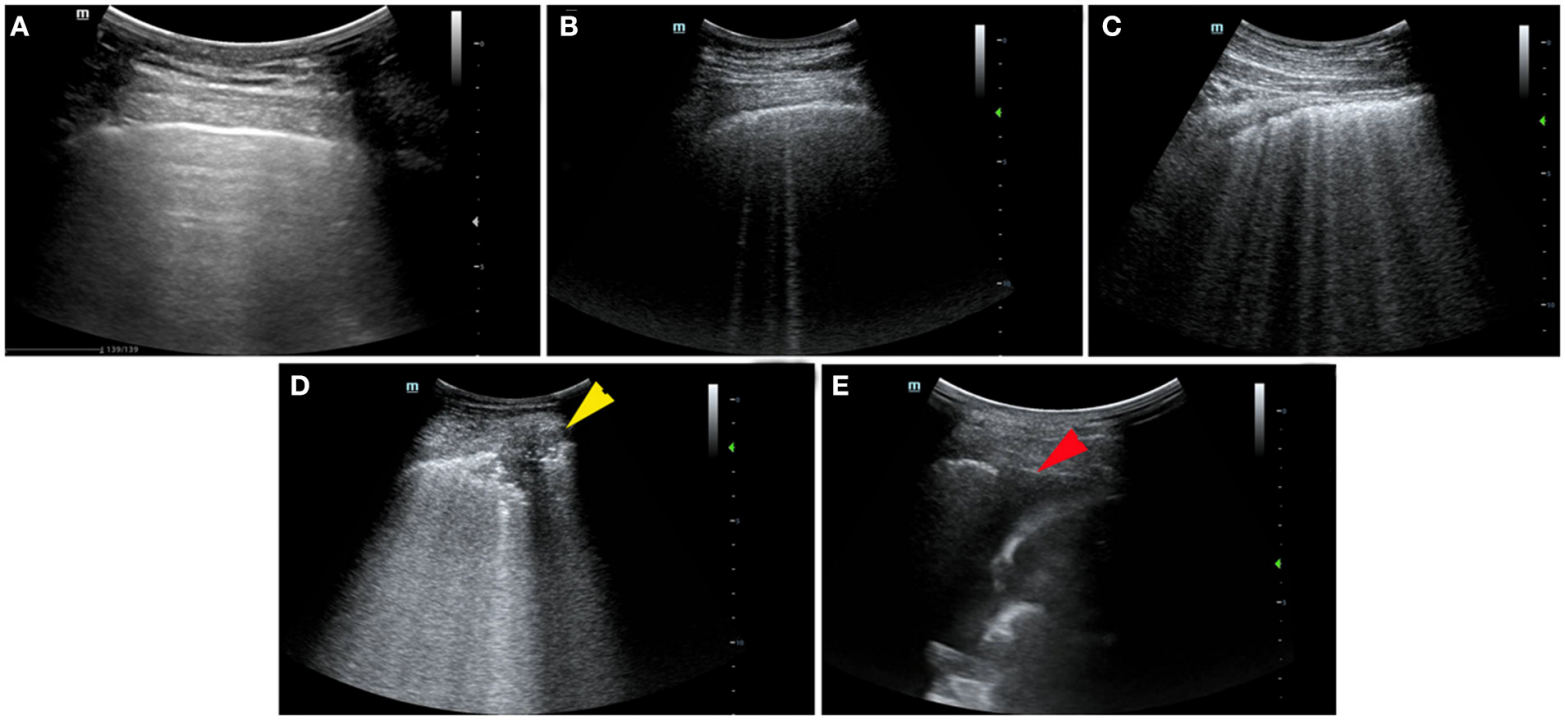
Ultrasonographic features and lung ultrasound (LUS) score in patients with coronavirus disease 2019 (COVID-19). **(A)** Normal: the presence of A lines beyond the pleural line characterizes mornal pulmonay aeration, LUS score: 0. **(B)** B1 line: the presence of multiple vertical B lines (comet tails) with well-defined spacing regularly spaced B lines 7 mm apart, LUS score: 1. **(C)** B2 line: the presence of coalescent B lines <3 mm apart, LUS score: 2. **(D)** Lung consolidation: the presence of a tissue pattern (yellow arrowhead), LUS score: 3. **(E)** Pleural effusion at costophrenic angle (red arrowhead).

Examples of ultrasound findings including the patterns of B lines, consolidations, pleural line abnormalities, pleural effusion, and the lesion distribution are shown in Figure 1.

### Lung Ultrasound Score

LUS score was determined based on four lung patterns (Supplementary Table 1): N = 0, B1 = 1, B2 = 2, and C = 3 as described previously (7):

- N pattern—normal aeration: A lines or <3 isolated B lines;
- B1 pattern—moderate loss of lung aeration: a clear number of multiple visible B lines with horizontal spacing between adjacent B lines ≤7 mm (B1 lines);
- B2 pattern—severe loss of lung aeration: multiple B lines fused together with horizontal spacing between adjacent B lines ≤3 mm, including “white lung” (B2 lines); and
- C pattern—complete loss of aeration: pulmonary consolidation, presence of tissue pattern accompanied by static or dynamic air bronchograms.

Global LUS score was calculated by summing the scores of all 12 lung regions (ranging from 0 to 36). An adjusted composite score, antero-lateral score, was also derived by summing the anterior and lateral regional scores (range from 0 to 24) (5, 7).

### Repeatability and Reproducibility of Lung Ultrasound Score

Intra- and interobserver variability of global LUS score was assessed in 30 randomly selected subjects by repeat measurements on the same images 1 month apart by two observers. Bland–Altman plots were produced.

### Statistical Analysis

Demographic, clinical, and outcome variables were presented as percentages for categorical variables and as medians with interquartile ranges (IQRs) for continuous variables. The Mann–Whitney *U*-test was used to compare LUS scores between survivors and non-survivors.

Receiver operating characteristic (ROC) curves for death were drafted for global and anterolateral score. The area under the receiver operating characteristic curves (AUCs) was calculated to determine the diagnostic accuracy for death. The optimal cutoffs were determined as the highest Youden's index (sensitivity + specificity – 1).

Kaplan–Meier curves were used to examine cumulative death rate, and differences between groups were tested using a log rank test. Univariate and multivariate Cox regression analysis was performed to identify potential predictors of death. Multivariate models were constructed to assess the prognostic utility of global and anterolateral scores, incorporating covariables that were significant (*p* < 0.05) in the univariate analysis. All statistical analyses were performed using SPSS version 25 (SPSS Inc. Chicago, Illinois).

## Results

### Patient Characteristics

**Figure 2:**
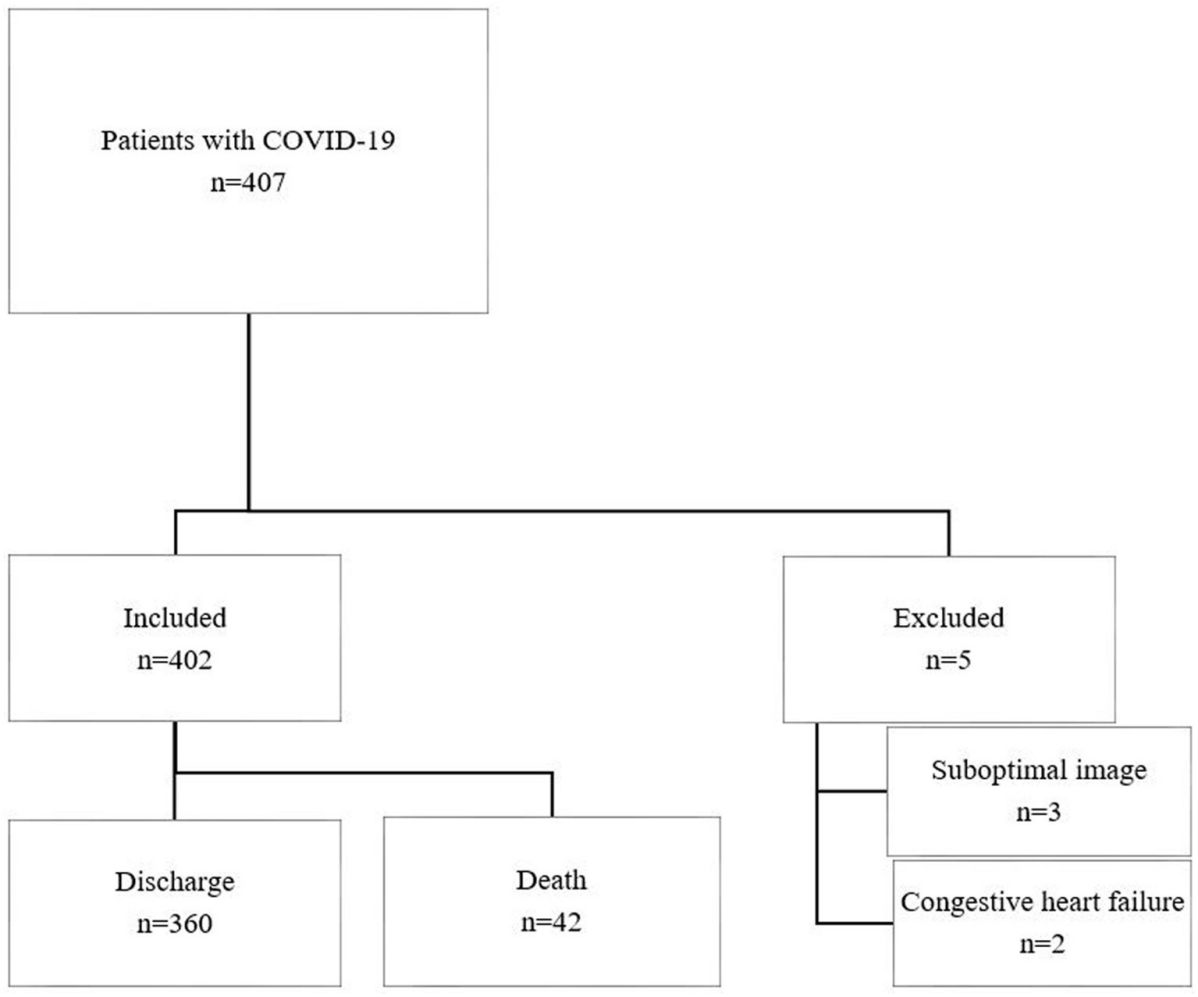
Study flow chart.

A total of 407 patients with COVID-19 meeting the inclusion criteria were recruited, of whom 5 were excluded due to suboptimal LUS images (*n* = 3) and congestive heart failure (*n* = 2) (Figure 2). Four hundred two patients were included in the final analysis, of whom 42 died with median time to death 21 (IQR, 14–29) days. Cause of death was recorded as multiorgan failure (42.9%), respiratory failure (26.1%), cardiac (9.5%), septic shock (9.5%), unknown (7.1%), and stroke (4.8%). Baseline characteristics are summarized in Table 1. Non-survivors were older and more male gender compared to survivors. There was a higher prevalence of preexisting conditions including hypertension, coronary heart disease (CHD), and malignancy in non-survivors compared to survivors.

**Table 1:** Patient characteristics.

		**No. (%)**			***p*-value**	**No. (%)**		***p*-value**
		**Total (*N* = 402)**	**Survivors (*N* = 360)**	**Non-survivors (*N* = 42)**		**Global LUS Score <15 (*N* = 310)**	**Global LUS Score ≥15 (*N* = 92)**	
**Age, median (IQR), years**		63 (52–70)	62 (52–69)	69 (61–77)	<0.001	61 (51–68)	69 (61–77)	<0.001
**Age distribution**		–	–	–	<0.001	–	–	<0.001
	20–40 years	39 (9.7)	39 (10.8)	0		36 (11.6)	4 (4.3)	
	40–60 years	125 (31.1)	124 (34.5)	1 (2.4)		112 (36.1)	18 (19.6)	
	≥60 years	238 (59.2)	197 (54.7)	41 (97.6)		162 (52.3)	70 (76.1)	
**Sex**		–	–	–	0.002	–	–	0.002
	Female	210 (52.2)	199 (55.3)	11 (26.2)		175 (56.5)	35 (38.0)	
	Male	192 (47.8)	161 (44.7)	31 (73.8)		135 (43.5)	57 (62.0)	
**Clinical presentation**								
	Fever	395 (98.2)	353 (98.0)	42 (100)	0.36	304 (98.1)	91 (98.9)	0.93
	Dry cough	279 (69.4)	246 (68.3)	33 (78.6)	0.17	209 (67.4)	70 (76.1)	0.11
	Headache	23 (5.7)	18 (5.0)	5 (11.9)	0.14	14 (4.8)	9 (9.8)	0.06
	Sore throat	45 (11.1)	42 (11.7)	3 (7.1)	0.53	31 (10.0)	14 (15.3)	0.16
	Myalgia	135 (33.6)	116 (32.2)	19 (45.2)	0.09	97 (31.3)	38 (41.3)	0.07
	Fatigue	131 (32.6)	115 (31.9)	16 (38.1)	0.42	100 (32.3)	31 (33.7)	0.80
	Dyspnea	124 (30.8)	104 (23.2)	20 (34.8)	0.01	72 (23.2)	32 (34.8)	0.03
	Rhinorrhea	43 (10.7)	35 (9.4)	8 (21.4)	0.11	32 (18.6)	11 (16.8)	0.66
	Nausea and vomiting	26 (6.5)	24 (6.7)	2 (4.8)	0.89	23 (7.4)	3 (3.3)	0.15
	Diarrhea	51 (12.7)	47 (13.1)	4 (9.5)	0.52	37 (11.9)	14 (15.2)	0.41
**Comorbidities**								
	Hypertension	97 (24.1)	80 (22.2)	17 (40.5)	0.009	64 (20.6)	33 (35.9)	0.003
	Coronary heart disease	50 (12.4)	35 (9.7)	15 (35.7)	<0.001	30 (9.7)	20 (21.7)	0.002
	Arrhythmia	10 (2.5)	9 (2.5)	1 (2.4)	1.00	8 (2.6)	2 (2.2)	1.00
	Diabetes	40 (10.0)	36 (10.0)	4 (9.5)	1.00	27 (8.7)	13 (14.1)	0.13
	Cerebrovascular disease	12 (3.0)	9 (2.5)	3 (7.1)	0.23	6 (1.9)	6 (6.5)	0.06
	Chronic pulmonary Disease	15 (3.7)	11 (3.1)	4 (9.5)	0.01	9 (2.9)	6 (6.5)	0.11
	Chronic liver disease	17 (4.2)	15 (4.2)	2 (4.8)	1.00	12 (3.9)	5 (5.4)	0.72
	Chronic kidney disease	5 (1.2)	4 (1.1)	1 (2.4)	1.00	3 (1.0)	2 (2.2)	0.70
	Malignancy	25 (6.2)	18 (5.0)	7 (16.7)	0.009	14 (4.5)	11 (12.0)	0.01
**Clinical outcome**		–	–	–	<0.001	–	–	<0.001
Discharged		360 (89.6)	360 (100)	0		305 (98.4)	55 (59.8)	
Died		42 (10.4)	0	42 (100)		5 (1.6)	37 (40.2)	
**ARDS**		85 (21.1)	43 (11.9)	42 (100)	<0.001	17 (5.5)	68 (73.9)	<0.001
**ICU admission**		79 (19.7)	38 (10.5)	41 (97.6)	<0.001	15 (4.8)	64 (69.6)	<0.001
**Mechanical Ventilation**		76 (18.9)	36 (10.0)	40 (95.2)	<0.001	13 (4.2)	63 (68.5)	<0.001
**Days from admission to ultrasonic examination, median (IQR), days**		3 (2–5)	3 (2–5)	3 (1–4)	0.44	3 (2–5)	3 (2–5)	0.32
**Length of hospital stay, median (IQR), days**		27 (20–39)	28 (21–40)	23 (15–31)	0.002	27 (20–40)	27 (19–37)	0.88

*Global LUS score: summing the scores of all 12 lung regions (two anterior, two lateral, and two posterior thoracic regions) (ranging from 0 to 36)*.

*ARDS, acute respiratory distress syndrome; ICU, intensive care unit*.

### Laboratory Findings

Laboratory data on hospital admission are summarized in Table 2. Overall, non-survivors had significant worse laboratory results, including increased white blood cell count, prothrombin time, activated partial thromboplastin time, D-dimer, aspartate aminotransferase, total bilirubin, blood urea nitrogen, creatinine, hypersensitive troponin I (hs-Tnl), lactate dehydrogenase (LDH), creatine kinase–myocardial band (CK-MB), hypersensitive C-reactive protein (CRP), and procalcitonin and decreased lymphocyte count, platelet count, hemoglobin, total protein, and albumin (all *p* < 0.05) compared to survivors. Patients with a higher global LUS score (>15) had significant worse laboratory results, in particular, significantly increased D-dimer and CRP compared to those with a global LUS score <15.

**Table 2:** Laboratory findings.

		**Median (IQR)**			***p*-value**	**Median (IQR)**		***p*-value**
		**Total (*N* =402)**	**Survivors (*N* = 360)**	**Non-survivors (*N* = 42)**		**Global LUS Score <15 (*N* = 310)**	**Global LUS Score ≥15 (*N* = 92)**	
**Blood count**								
	WBC count, × 109/L	5.94 (4.73–7.56)	5.85 (4.62–6.87)	6.99 (4.98–10.51)	0.045	5.85 (4.62–6.87)	6.99 (4.98–10.51)	<0.001
	Lymphocyte count, × 109/L	1.49 (1.11–1.87)	1.45 (1.09–1.85)	0.45 (0.28–0.78)	<0.001	1.60 (1.25–1.96)	0.97 (0.45–1.38)	<0.001
	Platelet count, × 109/L	205 (160–250)	210 (167–255)	140 (92–208)	<0.001	211 (168–256)	179 (139–223)	<0.001
	Hemoglobin, g/dl	120 (107–132)	121 (109–132)	104 (92–124)	0.001	122 (112–134)	107 (95–124)	<0.001
**Coagulation function**								
	PT, s, (*n* = 384)	13.0 (12.4–13.8)	12.9 (12.4–13.6)	15.8 (13.9–18.4)	<0.001	12.9 (12.4–13.6)	13.8 (12.8–16.2)	<0.001
	APTT, s, (*n* = 384)	37.1 (34.5–41.7)	36.7 (34.2–40.4)	47.9 (39.3–58.4)	<0.001	36.5 (34.2–40.3)	40.5 (35.1–49.5)	<0.001
	D-dimer, mg/L, (*n* = 384)	0.44 (0.22–1.22)	0.39 (0.21–0.93)	3.08 (1.36–8.00)	<0.001	0.37 (0.20–0.84)	1.10 (0.39–3.01)	<0.001
**Blood biochemistry**								
	TP, g/L	66.3 (62.7–70.2)	66.7 (63.5–70.6)	59.5 (54.8–65.4)	<0.001	66.7 (63.6–70.5)	64.3 (57.7–68.4)	0.003
	Albumin, g/L	38.6 (35.0–41.5)	39.2 (36.3–41.8)	26.9 (24.4–30.1)	<0.001	39.6 (365. −41.9)	33.7 (27.0–38.2)	<0.001
	ALT, U/L	28 (19–47)	29 (19–46)	37.0 (22–70)	0.06	29.0 (19.5–46.0)	26.0 (18.0–47.0)	0.08
	AST, U/L	24 (19–32)	23 (19.0–31)	42 (29–75)	0.01	23.0 (18.0–30.5)	31 (22.0–45.0)	0.02
	TB, μmol/L	10.4 (7.8–13.7)	10.0 (7.7–13.1)	14.7 (9.5–28.8)	0.002	10.2 (7.8–13.2)	11.0 (7.5–15.4)	0.05
	Sodium, mmol/L	139.8 (138.5–141.6)	139.7 (138.5–141.3)	141.5 (138.6–144.3)	0.05	139.8 (138.7–141.4)	139.8 (137.5–142.5)	0.98
	Potassium, mmol/L	4.15 (3.90–4.37)	4.16 (3.93–4.37)	3.96 (3.55–4.39)	0.73	4.17 (3.94–4.37)	4.10 (3.79–4.40)	0.58
	BUN, mmol/L, (*n* = 382)	4.92 (3.90–6.01)	4.75 (3.84–5.69)	10.61 (6.85–18.48)	<0.001	4.70 (3.87–5.70)	5.65 (4.23–10.52)	<0.001
	Creatinine, μmol/L	64.3 (53.8–77.0)	63.8 (53.9–75.5)	76.9 (50.7–140.3)	0.024	63.5 (54.3–75.7)	68.7 (50.3–91.0)	0.05
	hs-cTnI, pg/mL, (*n* = 382)	3.3 (1.7–12.1)	2.6 (1.6–6.5)	100.6 (29.3–407.4)	<0.001	2.50 (1.53–5.18)	15.4 (4.12–98.05)	<0.001
	LDH, U/L	180 (153–228)	174 (151–206)	393 (278–670)	<0.001	174 (150–206)	216 (166–365)	0.001
	CK-MB, U/L (*n* = 347)	0.9 (0.4–9.0)	0.8 (0.4–7.0)	21.6 (9.0–34.3)	0.008	0.8 (0.4–8.0)	1.9 (0.6–21.1)	0.03
**Infection-related biomarkers**								
	CRP, mg/L, (*n* = 370)	3.03 (0.72–10.4)	2.43 (0.62–5.92)	90.19 (53.7–125.8)	<0.001	2.37 (0.59–5.8)	24.93 (2.21–105.6)	<0.001
	PCT, ng/ml, (*n* = 370)	0.06 (0.04–0.13)	0.06 (0.04–0.11)	0.38 (0.14–1.51)	<0.001	0.06 (0.04–0.10)	0.07 (0.07–0.43)	0.03

*WBC, white blood cell; PT, prothrombin time; APTT, activated partial thromboplastin time; TP, total protein; ALT, alanine transaminase; AST, aspartate aminotransferase; TB, total bilirubin; BUN, blood urea nitrogen; hs-cTnI, hypersensitive troponin I; LDH, lactate dehydrogenase; CK-MB, creatine kinase–MB; CRP, hypersensitive C-reactive protein; PCT, procalcitonin*.

### Lung Ultrasound Findings and Lung Ultrasound Score

**Figure 3:**
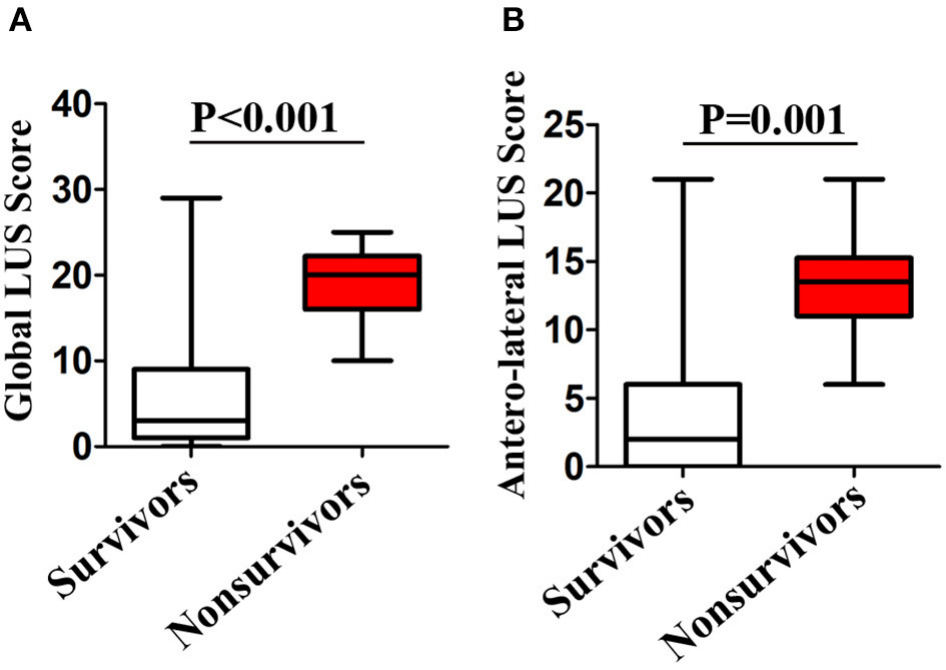
Box plots representation of **(A)** global and **(B)** anterolateral lung ultrasound (LUS) scores in survivors and non-survivors.

Lung ultrasound was performed within a median of 3 (IQR, 2–5) days from hospital admission. Lung ultrasound findings are shown in Table 3. Eighty-four patients (20.9%) had normal LUS. The presence of B lines was the most common finding (318/402, 79.1%), followed by pleural line abnormalities (137/402, 31.8%) and consolidation (117/402, 25.6%). Pleural effusions were detected in 36 (8.2%) patients. Compared to survivors, non-survivors were more likely to have B2 lines, pleural line abnormalities, pulmonary consolidation, and pleural effusion, but there was no difference in the presence of B1 lines. All non-survivors had bilateral involvement. Survivors had significantly lower global and anterior–lateral LUS scores compared to non-survivors (Figure 3). Findings of each of 12 lung regions are shown in Supplementary Figure 2. Regional LUS scores including anterior, lateral, and posterior scores are presented in Supplementary Figure 3. Bland–Altman plots for intra- and interobserver variability of global LUS score are shown in Supplementary Figure 4. All repeated measures were within 1.96 × standard deviation of the mean, which suggested a good reproducibility of global LUS score.

**Table 3:** Lung ultrasound findings.

		**No. (%)**			***p*-value**
		**Total (*N* = 402)**	**Survivors (*N* = 360)**	**Non-survivors (*N* = 42)**	
**Normal baseline lung ultrasound**		84 (20.9)	84 (23.3)	0	<0.001
**Abnormal baseline lung ultrasound**		318 (79.1)	276 (76.7)	42 (100)	
**Characteristics of lung ultrasound**					
	B line	318 (79.1)	276 (76.7)	42 (100)	<0.001
	B1 line	236 (58.7)	210 (58.3)	26 (61.9)	0.66
	B2 line	213 (51.5)	171 (45.8)	42 (100)	<0.001
	Pleural line abnormalities	137 (31.8)	103 (26.4)	34 (78.6)	<0.001
	Pulmonary consolidation	117 (25.6)	83 (20.6)	34 (69.0)	<0.001
	Pleural effusion	36 (8.2)	18 (4.4)	18 (40.5)	<0.001
**Distribution at baseline ultrasound**					<0.001
	Right lung	63 (15.7)	63 (17.5)	0	
	Left lung	30 (7.5)	30 (8.3)	0	
	Bilateral lungs	223 (55.5)	181 (50.3)	42 (100)	
	Abnormalities at lung region				
	Left anterior superior	129 (32.1)	93 (25.8)	36 (85.7)	<0.001
	Left anterior inferior	112 (28.4)	74 (20.6)	38 (90.5)	<0.001
	Left lateral superior	128 (31.8)	100 (27.8)	28 (66.7)	<0.001
	Left lateral inferior	153 (38.1)	112 (31.1)	41 (97.6)	<0.001
	Left posterior superior	111 (27.6)	81 (22.5)	30 (71.4)	<0.001
	Left posterior inferior	156 (38.8)	125 (34.7)	31 (73.8)	<0.001
	Right anterior superior	139 (34.5)	108 (30.0)	31 (73.8)	<0.001
	Right anterior inferior	138 (34.3)	102 (28.3)	36 (85.7)	<0.001
	Right lateral superior	129 (32.1)	103 (28.6)	26 (61.9)	<0.001
	Right lateral inferior	160 (39.8)	120 (33.3)	40 (95.2)	<0.001
	Right posterior superior	142 (35.3)	107 (29.7)	35 (83.3)	<0.001
	Right posterior inferior	150 (37.3)	130 (36.1)	20 (47.6)	0.14
**Global LUS score, median (IQR)**		4 (1–13)	3 (1–9)	20 (18–23)	<0.001
**Anterolateral LUS score, median (IQR)**		2 (0–8)	5 (0–9)	14 (11–15)	0.001

### Prediction of Mortality by LUS Global and Anterolateral Score

**Figure 4:**
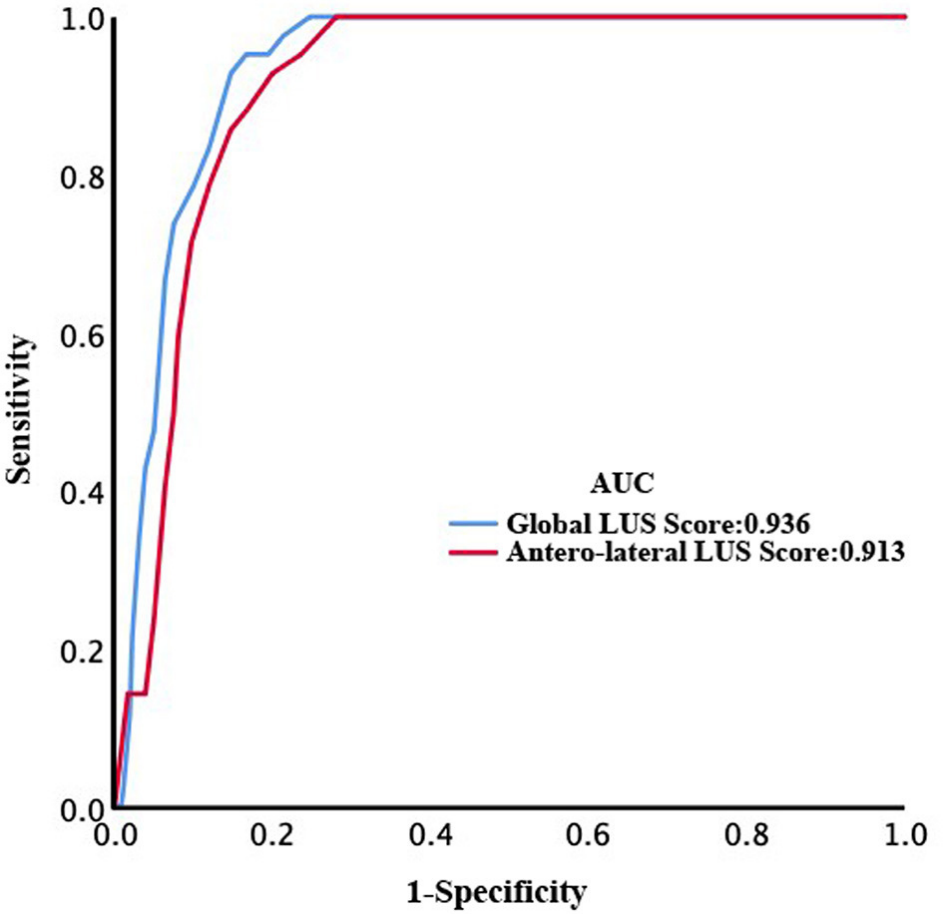
Receiver operating characteristic (ROC) curves of global and anterolateral lung ultrasound (LUS) score for prediction of death.

After a median of 27 (IQR, 20–39) days of follow-up, 42 patients died. ROC curve analyses of global and anterolateral LUS score for predicting mortality are shown in Figure 4. The area under the curve were 0.936 and 0.913 for global and anterolateral LUS score, respectively. A cutoff value of 15 for global LUS score had a sensitivity of 92.9% and specificity of 85.3% for prediction of death, and a cutoff value of 9 for anterolateral LUS score had a sensitivity of 88.1% and specificity of 83.3%. Clinical characteristics and laboratory findings dichotomized according to global LUS score optimal value of 15 are shown in Tables 1, 2.

**Figure 5:**
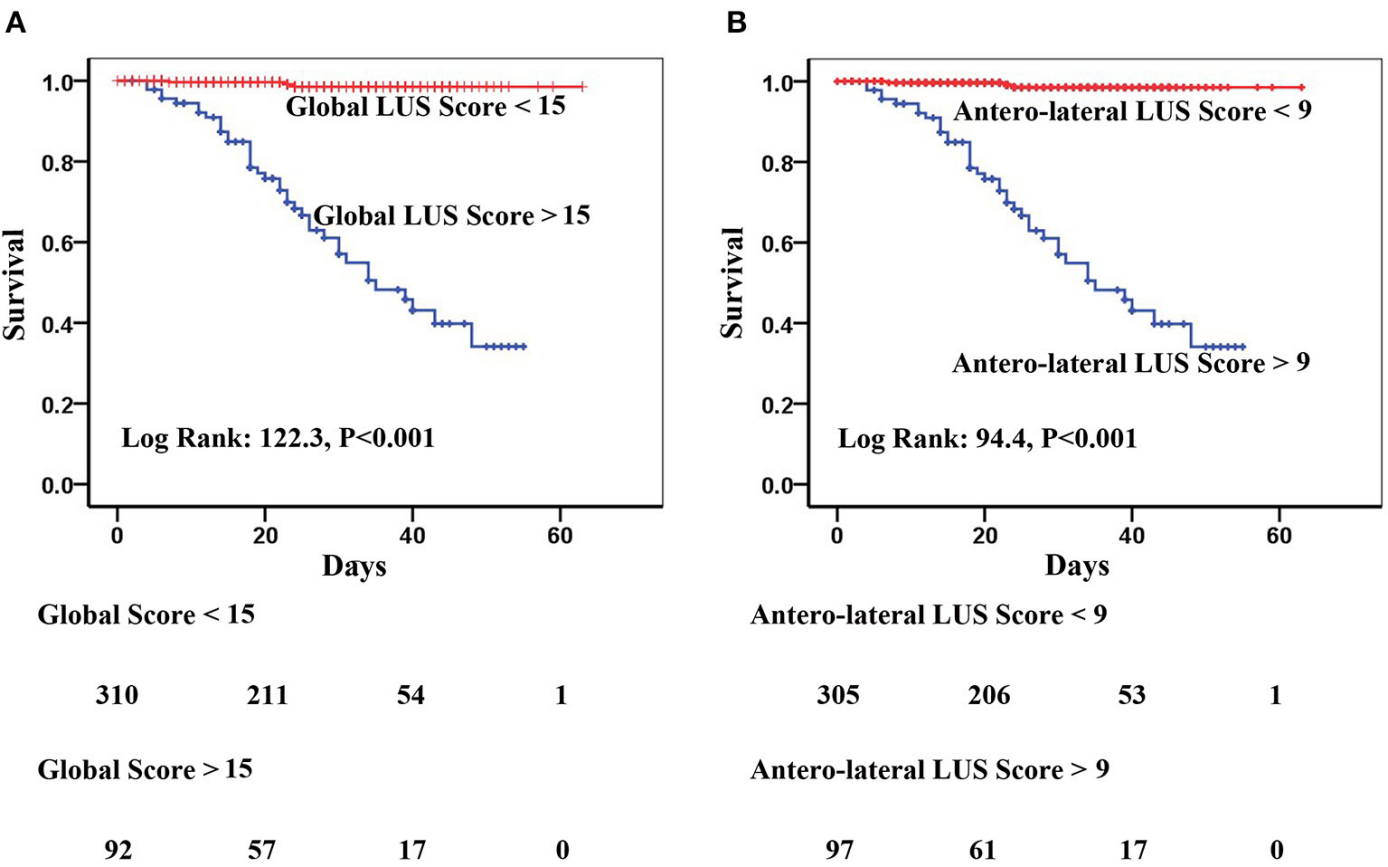
Kaplan–Meier curves of **(A)** global lung ultrasound (LUS) score with optimal cutoff value of 15 and **(B)** anterolateral LUS score with optimal cutoff value of 9 for prediction of in-hospital mortality.

Kaplan–Meier analysis showed that both global and anterolateral LUS scores were strong predictors of death (Figure 5). When global LUS score was >15, 37/92 (40.2%) patients died compared to only 5/310 (1.6%) death in those with a global LUS score <15. When patients were dichotomized by anterolateral LUS score of 9, there were 36/97 (37.1%) deaths in patients with a high score compared to 6/305 (2.0%) deaths in those with a low anterolateral score.

On univariate Cox regression analysis, age, male gender, malignancy, CHD, CRP, hs-cTnl, CK-MB, D-dimer, global LUS score, and anterolateral LUS score were significantly associated with mortality (Table 4). In multivariate model 1, considering global LUS score together with other significant predictors in the univariate model, age, male sex, CRP, CK-MB, and global LUS score [hazard ratio (HR), 1.08; 95%CI, 1.01–1.16, *p* = 0.03) remained as a significant predictor. In multivariate model 2, when anterolateral LUS score was tested with other variables, the predictive power of anterolateral LUS score did not remain significant.

**Table 4:** Univariate and multivariate Cox regression.

	**HR**	**CI (95%)**	***p***	**HR**	**CI (95%)**	***p***	**HR**	**CI (95%)**	***p***
	**Univariate**			**Model 1**			**Model 2**		
Age	1.04	1.01–1.07	**0.005**	1.05	1.00–1.10	**0.04**	1.05	1.00–1.09	**0.05**
Male sex	0.33	0.17–0.67	**0.002**	0.31	0.11–0.89	**0.03**	0.34	0.12–0.92	**0.03**
Hypertension	0.55	0.30–1.03	0.06						
Malignancy	0.32	0.14–0.72	**0.006**	0.57	0.18–1.81	0.34	0.57	0.18–1.82	0.34
CHD	0.28	0.15–0.52	**<0.001**	0.99	0.45–2.18	0.99	0.93	0.43–2.02	0.85
CRP	2.58	1.99–3.35	**<0.001**	1.60	1.17–2.20	**0.004**	1.69	1.23–2.31	**0.001**
hs-cTnl	1.82	1.61–2.04	**<0.001**	1.11	0.90–1.37	0.34	1.17	0.95–1.44	0.14
CK-MB	2.11	1.75–2.54	**<0.001**	1.47	1.09–1.99	**0.01**	1.54	1.13–2.08	**0.006**
D-Dimer	2.75	2.08–3.65	**<0.001**	1.19	0.83–1.69	0.34	1.17	0.82–1.66	0.38
Global LUS score	1.20	1.15–1.26	**<0.001**	1.08	1.01–1.16	**0.03**			
Anterolateral LUS score	1.23	1.17–1.29	**<0.001**				1.04	0.96–1.13	0.34
C-index					**0.995**			**0.994**	

*CHD, coronary heart disease; CRP, C-reactive protein; hs-cTnl, hypersensitive troponin I; CK-MB, creatine kinase–myocardial band*.

*Global LUS score: summing the scores of all 12 lung regions (two anterior, two lateral, and two posterior thoracic regions) (ranging from 0 to 36). The values in bold represent statistical differences in data*.

## Discussion

Our data suggested that global LUS score was a predictor of in-hospital mortality independent of age, gender, comorbidities, and biochemical markers and was superior to LUS anterolateral score. The optimal threshold of 15 for global LUS score and 9 for anterolateral LUS score were in line with those derived from previous investigations (5, 8). These findings supported the clinical utility of LUS in patients with COVID-19 (7, 9) given its ease of use at point of care, low cost, lack of radiation exposure, and ready combination with other components of critical care ultrasonography (10, 11).

LUS features in patients with COVID-19 in our study manifested as multiple lesions, various types of B lines, irregularly pleural lines, and subpleural consolidations. B lines presented in 79.1% patients. B2 lines and consolidations were more common in non-survivors than in survivors. Pleural effusion, pleural thickening, and pneumothorax were less common in COVID-19 patients, which were consistent with the latest autopsy report (12) that COVID-19 patients presented with acute interstitial lung disease.

Bass et al. showed that LUS had high sensitivity for detection of interstitial and alveolar–interstitial lung disease with peripheral distribution (13). Consistent with these features, our findings suggested that global LUS score was highly predictive of death in COVID-19 and independent of other previously identified predictors. Non-survivors in our study were older and more male with higher prevalence of preexisting conditions including hypertension, CHD, and malignancy and higher levels of cardiac injury and systematic inflammation markers than survivors, which were in consistence with previous studies (14).

Another interesting finding of our study was that when the posterior regions were excluded, the predictive power of anterolateral LUS score disappeared in the multivariate cox regression model. This finding was consistent with chest CT findings that the most commonly involved lung segments in patients with COVID-19 were the dorsal segment of the right lower lobe, the posterior basal segment of the right lower lobe, the lateral basal segment of the right lower lobe, and the dorsal segment and the posterior basal segment of the left lower lobe (15). Despite some studies showing that the posterior regions had the lowest diagnostic accuracy (5), scores from these regions could play an important role in risk stratification. In the present study, lung lesions were mainly located in the right lateral inferior area (39.8%), left lateral inferior area (38.1%), left posterior inferior area (38.8%), and right posterior inferior area (37.3%) (the lower posterior and lateral segments of the lungs). This finding also supported that the potential benefit of prone position in patients affected with COVID-19 acute respiratory distress syndrome (ARDS) due to a more even distribution of the gas–tissue ratios along the dependent–non-dependent axis and a more homogeneous distribution of lung stress and strain (16).

Although anterolateral LUS score had less predive power compared to global LUS score, it may still play an important role particularly in patients on ICU.

### Clinical Implications

COVID-19 as a global pandemic imposes a huge burden on medical systems. Early quantification of patients with severe lung involvement may be critical for optimization of treatment and management. LUS as a non-invasive and cost-effective diagnostic tool can be performed rapidly, particularly in ICU. Severe studies have also demonstrated that echocardiography is a crucial tool in detecting cardiovascular complications (in particular on assessment of left and right ventricular function) and predicts poor prognosis in patients COVID-19 (17, 18). Combining LUS with echocardiography may add additional value to identify patients at higher risk of poor outcomes.

### Limitations

Our study has several limitations. First, mortality rate was relatively low, which limits the strength of our conclusion. Low mortality rate may be due to the fact that majority of patients in the present study were not in ICU, while this rate was similar to previously published data (19). Second, the follow-up period was relatively short, as majority of patients were discharged within 28 days from admission.

Although our findings suggested that LUS may add additional value in risk stratification, the strength of our conclusion may be limited by the nature of an observational study. There are several other limitations of LUS that cannot be ignored such as the requirement of special training to perform high-quality LUS, lack of evidence-based guidelines, the high risk of infection when performing LUS examination in patients with COVID-19 (20, 21).

Patients included in this study were recruited from three hardest-hit hospitals in Wuhan, and these patients may not represent the population in other areas. Finally, the relationship between LUS and lung CT was not explored, as the majority of patients did not have lung CT due to limited availabilities and the nature of infectious disease.

## Conclusion

Global LUS score as a semiquantitative measure of lung conditions is a powerful predictor of in-hospital mortality in patients with COVID-19 and may add additional value in patient monitoring and risk stratification.

## Data Availability Statement

The raw data supporting the conclusions of this article will be made available by the authors, without undue reservation.

## Ethics Statement

The studies involving human participants were reviewed and approved by Union Hospital, Tongji Medical College, Huazhong University of Science and Technology. Written informed consent for participation was not required for this study in accordance with the national legislation and the institutional requirements. Written informed consent was obtained from the individual(s) for the publication of any potentially identifiable images or data included in this article.

## Author Contributions

ZS, HW, HG, and MX conceived and designed the study. ZZ and JL contributed to the literature search. ZS, CC, SQ, YS, YD, WZ, MY, and LJ contributed to data collection. ZZ, CY, and HG contributed to data analysis. JW, YY, QL, and HG contributed to data interpretation. YX and RW contributed to the figures. ZS, HG, ZZ, and JL drafted the article. All authors contributed to the article and approved the submitted version.

## Conflict of Interest

The authors declare that the research was conducted in the absence of any commercial or financial relationships that could be construed as a potential conflict of interest.
