# Supplementary material for: Lung Ultrasound Score as a Predictor of Mortality in Patients With COVID-19
Source: Front Cardiovasc Med. 2021 May 25;8:633539. doi: 10.3389/fcvm.2021.633539 (PMC8185027; doi:10.3389/fcvm.2021.633539)
Supplement: Supplementary file 1 [file Data_Sheet_1.DOCX]

**Supplement table and Figures**

**Table S1 Calculation of Lung Ultrasound Score**

|  | **Aeration** | **Lung ultrasound appearance** |
| --- | --- | --- |
| **Score 0** | normal | A lines-max 2 B lines |
| **Score 1** | moderate loss | B1 lines (≥3 well-spaced B lines with horizontal spacing between adjacent B lines ≤ 7 mm) |
| **Score 2** | severe loss | B2 lines (multiple B lines fused with horizontal spacing between adjacent B lines ≤ 3 mm) |
| **Score 3** | complete loss | echoic lung tissue, accompanied by dynamic air bronchogram |

Global LUS was calculated by summing the highest score of each region (0-36)

Antero-lateral score was calculated by summing the anterior and lateral regional scores (0-24)

**
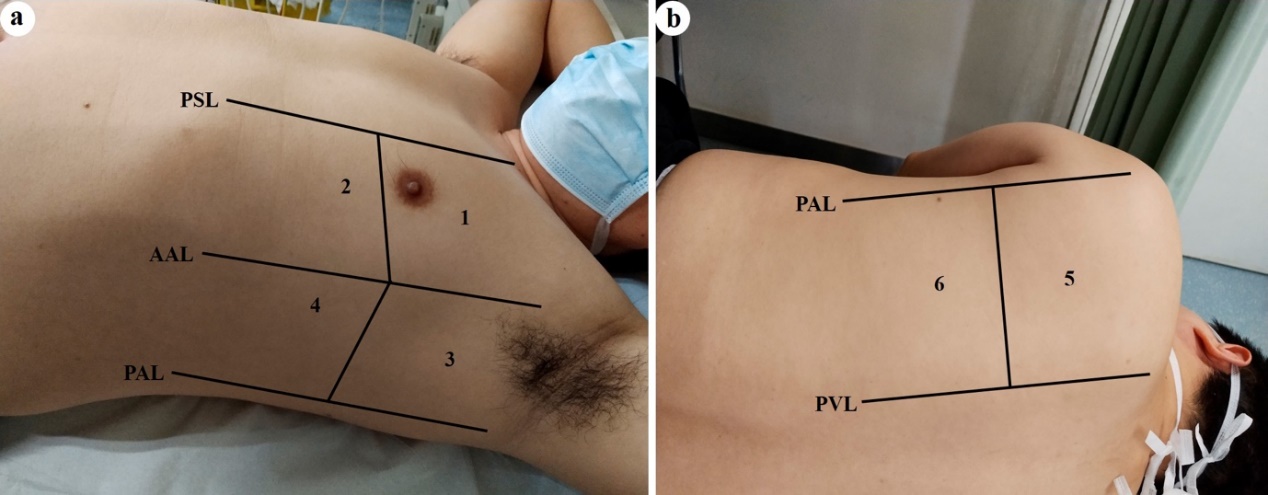
**

**Figure S1 Lung ultrasound regions – PSL: Parasternal line; AAL: Anterior axillary line; PAL: Posterior axillary line; PVL: Paravertebral line.**


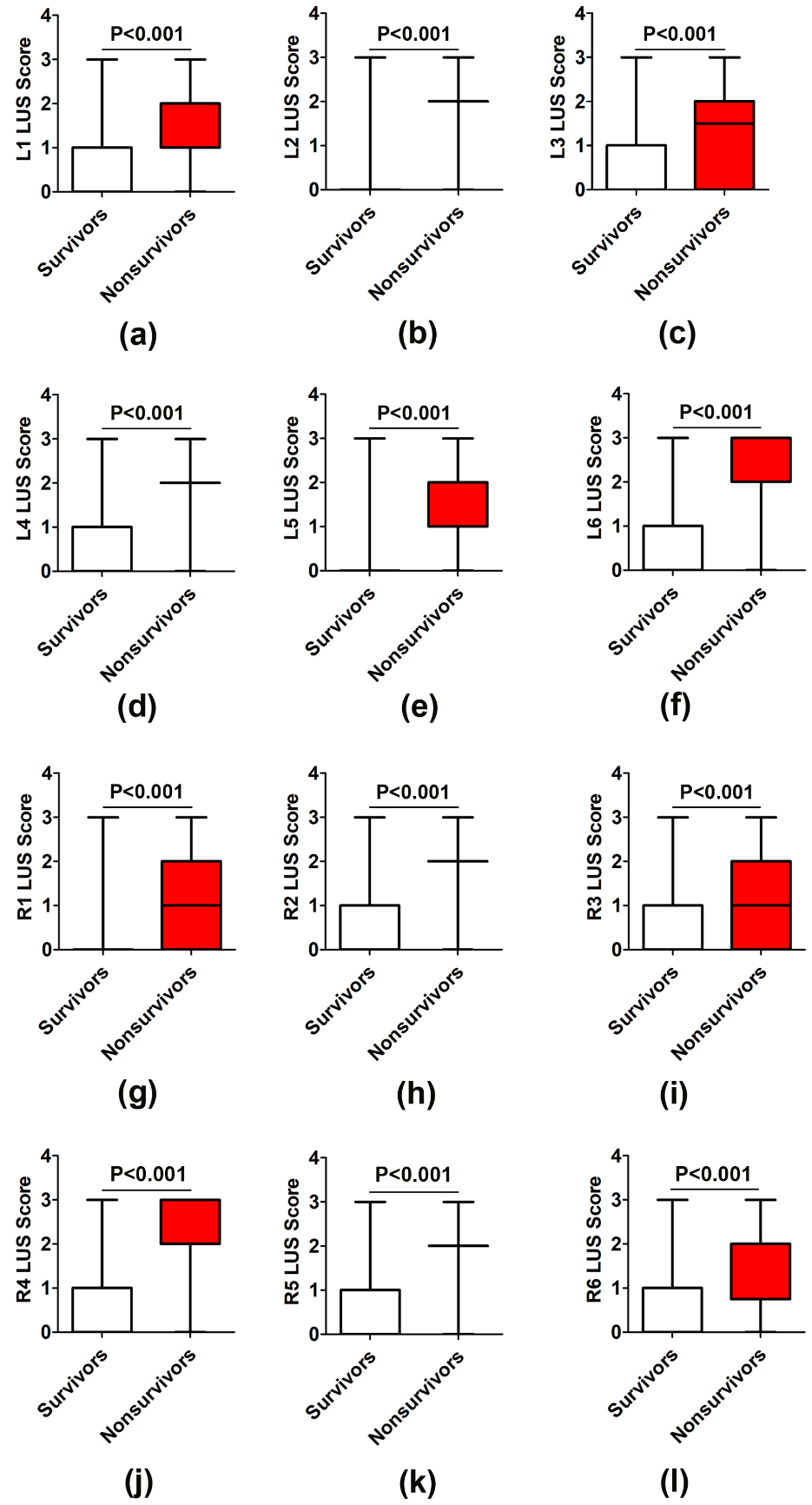


**Figure S2.** **LUS scores in each region**

(a) LUS score of left anterior superior,(b) LUS score of left anterior inferior, (c) LUS score of left lateral superior, (d) LUS score of left lateral inferior, (e) LUS score of left posterior superior, (f) LUS score of left posterior inferior, (g) LUS score of right anterior superior, (h) LUS score of right anterior inferior, (i) LUS score of right lateral superior, (j) LUS score of right lateral inferior, (k) LUS score of right posterior superior, (l) LUS score of right posterior inferior

L1: left anterior superior,L2: left anterior inferior,L3: left lateral superior,L4: left lateral inferior,L5: left posterior superior,L6: left posterior inferior,R1: right anterior superior,R2: right anterior inferior,R3: right lateral superior,R4: right lateral inferior,R5: right posterior superior,R6: right posterior inferior


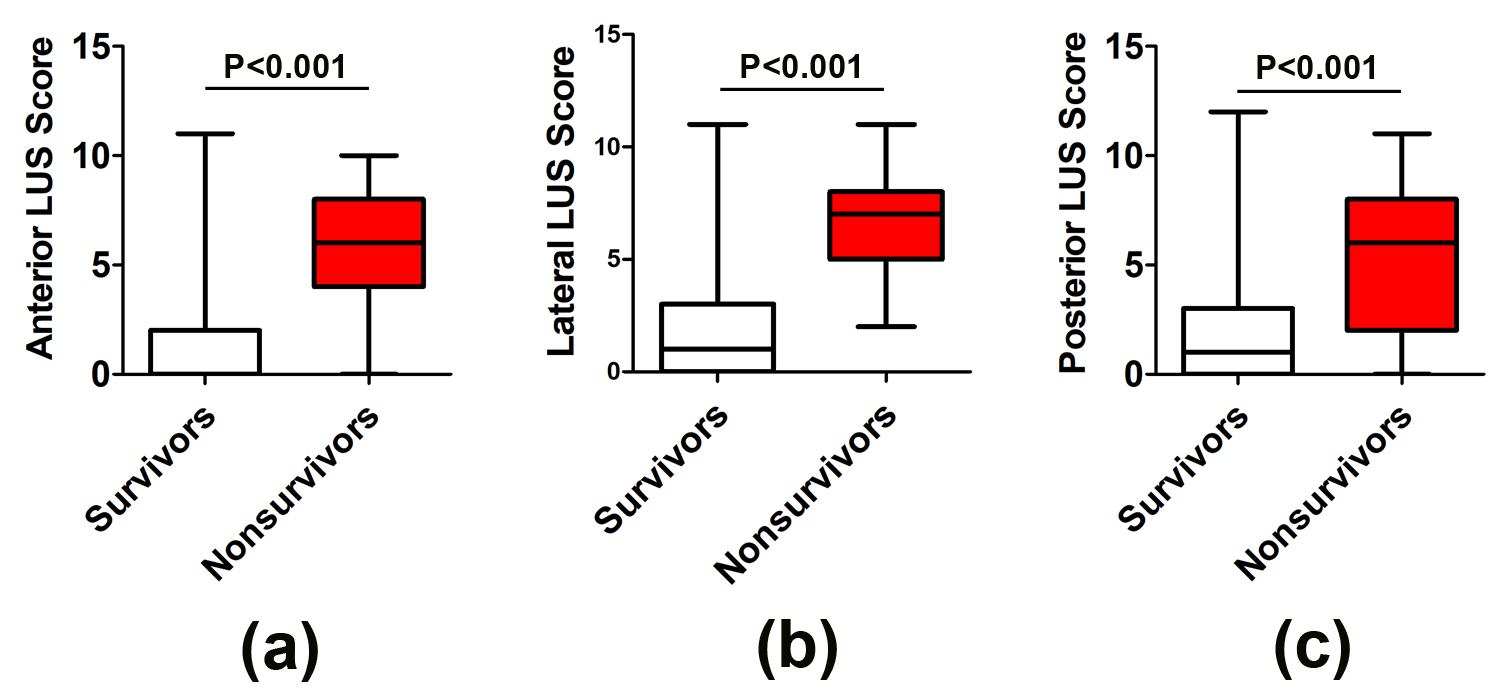


**Figure S3. Anterior, lateral,and posterior LUS scores**

1. Anterior LUS Scores
2. Lateral LUS Scores
3. Posterior LUS scores


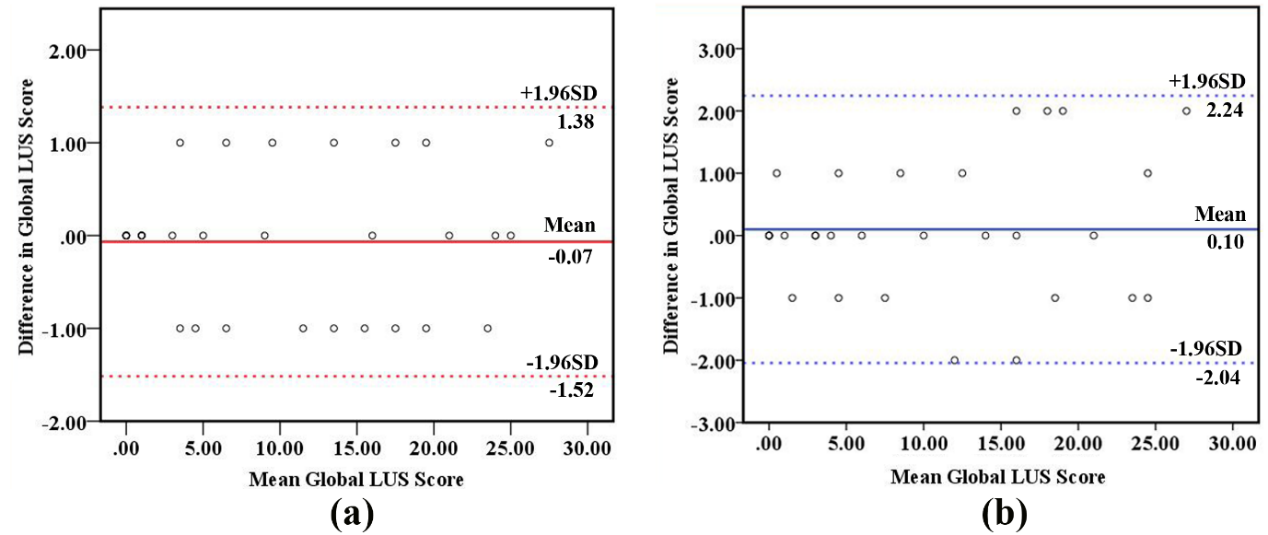


**Figure S4 Bland-Altman plots of measurement of global LUS score: a) intra- and b) inter-observer variability.**
